# Starters and non-starters soccer players in competition: is physical performance increased by the substitutions?

**DOI:** 10.1186/s13102-023-00641-3

**Published:** 2023-03-16

**Authors:** Alfonso Castillo-Rodríguez, José Luis González-Téllez, Antonio Figueiredo, José Luis Chinchilla-Minguet, Wanesa Onetti-Onetti

**Affiliations:** 1 Department of Physical Education and Sports, University of Granada, Granada, Spain; 2 Research Unit for Sport and Physical Activity, Faculty of Sport Sciences and Physical Education, University of Coimbra, Coimbra, Portugal; 3 Department of Didactics of Languages, Arts and Sport, University of Malaga, Boulevard Louis Pasteur s/n, 29011 Malaga, Spain; 4 Facultad de Educación, Universidad Internacional de la Rioja, Logroño, Spain

**Keywords:** Football, Role, Playing position, Distance, Global positioning system

## Abstract

**Background:**

Non-starters soccer players have a great role within the team, being indispensable to reduce fatigue, as well as to maintain and increase the team’s performance during the match.

**Objective:**

This study aimed to analyze the physical performance of the starter and non-starters players during competitive soccer matches.

**Methods:**

Twenty-two soccer players participated in this study, divided into two groups according to the role in the match (starters or non-starters). WIMU Global Positioning System devices were used in order to record physical performance metrics. Independent samples *t*-test and one-way ANOVA tests were performed to compare starters and non-starters, and the playing position, respectively, and two-way ANOVA test was perform with these factors too.

**Results:**

There were no differences in the main physical performance metrics between starters and non-starters players during competition, although there were differences in physical performance metrics according to the playing position. Midfielders performed highest distance per minute, player load, and distance covered between 12 and 21 km·h^− 1^ (p < .05). Finally, distance covered at speeds greater than 24 km·h^− 1^ was predicted by the playing role (starters and non-starters) and playing position factors with 88% of explained variance (*η*_*p*_^*2*^ = 0.772).

**Conclusion:**

The main findings of this study showed that non-starter players had a similar physical performance during competitive matches as the starter players for whom they are substituted. In addition, the playing position determined different physical performance, contributing in this manuscript that behavior and decision-making of the players could be affected by their position in the field. More studies are needed on non-starter player performance and contextual factors that could influence the physical responses of these players.

## Introduction

Soccer is the sport most practiced in the world, with a total of 250 million people [1]. It is characterized as an intermittent, high-intensity team sport, which makes high physical, tactical, and technical demands [2]. This sport is performed with high-intensity actions, i.e., accelerations, distances covered at high speed, sprints with and without change of directions, among others [3]. Each match lasts 90 min, plus extra time, where the high performance is required from the players, which decreases due to muscle fatigue [4, 5]. For these reasons, an appropriate distribution of the training loads could be beneficial for soccer players in order to perform a high physical level, according to the competition demands [6].

The sports success of a soccer team is related, among other factors, to the availability of players throughout a season. Some studies have shown that teams with fewer injuries and, therefore, more available players, completed the season with a higher final score in the general classification [7]. For these reasons, it is necessary to analyze the different external load metrics, i.e., distances covered, accelerations, metabolic power, sprints, among others, for appropriate monitoring and management of the load of both competitive matches and training during the season [8].

To maintain a high sports performance, teams, during the competition, have the possibility of making changes between starter and non-starter players, although in a limited way [9]. Traditionally, there were 3 changes, and currently, 5 changes can be made, although they still maintain 3 moments during the match. In this sense, non-starter players are decisive in keeping or improving a team’s sports performance [9]. The entry of these non-starter players in a match could be due to a decrease in the team performance, of a specific area or of a specific player. Several investigations have shown the need to know the positions that are most used for substitutions [10], as it could provide information to coaches about which positions on the field [11], according to the current game model are developing very high physical demands [12], which denotes at different times of the competition, a possible drop in performance [13]. This performance could be focused on physical, technical, or tactical factors, but could be conditioned by contextual metrics such as injuries, expulsions, minutes distribution, scoreboard, among others. [9, 14–16]. These non-starter players must offer an equal or superior performance during the competition, being the solution needed by coaches and the main reason for this choice, due to the limitation in the substitutions number [9]. In this case, there is a scarce literature that analyze the performance profile of non-starter players.

It would be necessary to know the timing and type of substitutions made in matches, and it would be of interest to quantify this to determine whether substitutions are made primarily for physical or tactical reasons [9]. Coaches, who make the decision to do substitutions, want there to be an effect, a change or an impact on the competition, whether physical or technical-tactical [16]. However, there is controversy about these impacts produced by substitute or non-starting players. On the one hand, those who enter the competition during the first half do not seem to make significant changes with respect to the starter players who already have accumulated fatigue [5]. In addition, those non-starters who entered the competition during the second half increased the total distance in the time ranges analyzed in previous research (absolute values), although they did not develop high-speed physical demands, compared to the starters [9]. Therefore, there is a need for further research and to really know if there is a physical reason in competition and if it occurs in that specific position or area of the field.

Once the problem approach has been explained, our initial hypothesis indicated that non-starter players develop similar or higher physical and physiological demands than the starter players who have been substituted. Therefore, the aim of this study was to analyze the physical performance in the competition according to the role of the starter and non-starter soccer players.

## Materials and methods

### Participants

Twenty-two soccer players (26.1 ± 5.7 years; 176.3 ± 3.6 cm; 73.3 ± 5.7 kg) participated in this study, classified into two groups, according to the role of starter or non-starter and within these, according to their playing position (goalkeeper excluded) [17]: full-back, center-back, midfielder and winger/forward, during the competitive match. The players belong to a senior team competing in the National competition in Spain. The evaluated team finished among the top four in the league. Data were collected from a total of 14 matches during the entire 2021/2022 season. As inclusion criteria, this study proposed that the non-starter players must enter the competition with at least 15 min before the end of the match. In addition, only starter players substituted will be incorporate to the database, however all players wore the Global Positioning System (GPS) device in the competition. Finally, 16 substitutions or events were recorded in the final database (Table 1), deleting 21 substitutions with less of 15 min of play (in total, 37 substitutions were studied initially). The main reason for deleting this data was because in most of the substitutions produced in the last 15 min, the team that made the substitutions was either winning the match or drawing away from home, so there was an interest in spent match time. The placement was done randomly among the members of the starting team, leaving 4 devices for the non-starter players. All players and the club were informed about the procedure and objective of this study and informed consent was obtained from both. It was reported that players would wear a harness with a wireless GPS device and would be tested if it was changed, so the player never knew if they would be tested. The researchers did not manipulate the independent variable and only the coach and the technical staff made the decision to make substitutions according to the needs of the competition. The Ethics Committee of the University of Granada (471/CEIH/2018) approved this study, and the guidelines of the Declaration of Helsinki (2013) were followed [18].

**Table 1:** Characteristics of the substitutions

ID	Playing position	Time played	Reason
1	Winger/Forward	34	Injury
2	Midfielder	59	Intensity increase
3	Midfielder	9	Injury
4	Full-back	60	Tactical change
5	Winger/Forward	19	Injury
6	Midfielder	74	Tactical change
7	Winger/Forward	57	Injury
8	Winger/Forward	74	Tactical change
9	Center-back	68	Intensity increase
10	Center-back	55	Tactical change
11	Center-back	75	Tactical change
12	Midfielder	61	Tactical change
13	Winger/Forward	40	Injury
14	Full-back	73	Intensity increase
15	Center-back	67	Intensity increase
16	Midfielder	66	Tactical change

### Instruments

Physical measurements were recorded using inertial devices (WIMU PRO™, RealTrack Systems, Almería, Spain). These devices had a high accuracy and reliability for metrics such as speed or distances, with a 1 GHz microprocessor [19]. In addition, they measured multiple metrics, thanks to this technology, such as accelerometers and location, which were frequently used to quantify physical demands in soccer [20, 21].

### Design and procedures

During the season, a total of 14 matches were recorded during the competitive period, between matchday 13 (October) and matchday 29 (April), using data from 16 substitutions for the study. In this period, all extracted data from the GPS of the starter and non-starter players was collected. Previously, the players trained with the devices for a month of training. For the analysis, we used the records that allowed us to compare between starter and non-starter players who were substituted in the same match and who both had the GPS device.

The data collection was varied because the days of the match ranged between Saturdays and Sundays, at different times, Saturdays from 5 to 9 pm and Sundays from 12 − 8 pm. Therefore, the temperature and degree of humidity presented was different. In no case were there match days of extreme or intense rain. Data was assessed both at home and away. The data collection procedure was always carried out by 2 researchers of the present study with extensive experience in research, in the use of GPS devices and in management with soccer teams.

### Physical performance metrics

Distance, running speed and metabolic metrics were analyzed, and all data were extracted from GPS devices and the individual body characteristics. Five categories of the distance run at different speeds ranges, measured in meters, were established according to criteria used in other studies [22]: low speed running distance (LSRD), from 6 to 12 km·h^− 1^; medium speed running distance (MSRD), from 12 to 18 km·h^− 1^; high speed running distance (HSRD), from 18 to 21 km·h^− 1^; very high speed running distance (VHSRD), from 21 to 24 km·h^− 1^; and sprint running distance (SPD), from 24 to 50 km·h^− 1^. In addition, another variable collected distances run at speeds of 0–6 km·h^− 1^. Data were also obtained in other metrics: distance (m), distance/time (m/min), accelerations (count), decelerations (count), accelerations (count/min), decelerations (count/min), explosive distance (m), explosive distance (m/min), player load (a.u.) and metabolic power (W/kg), in absolute and relative data.

### Statistical analyses

The statistical package SPSS for Windows version 23 (IBM SPSS Statistic, Chicago, USA) and Microsoft Office Excel (Microsoft Corp., Redmond, Washington, DC, IL, USA) were used. Considering a statistical power of 80%, a type 1 error or alpha of 0.05 and effect size of 0.82 (this is the value equivalent to a *R*^2^ = 0.45, which was the maximum prediction coefficient found in the literature for similar studies), we would need a minimum sample size of 12 substitutions.

The Kolmogorov-Smirnov test was analyzed, where the metrics were found to follow a normal distribution. *T*-tests were obtained for independent samples, because the players were different, in this case, a *t*-test was used, and the role of the game (starter or non-starter) as an independent variable for the means comparison. Subsequently, one-way ANOVA was performed in order to analyze the physical performance metrics with the playing position as factor (full-backs, center-backs, midfielders, and wingers/forwards). Finally, two-way ANOVA test was obtained using the playing role and playing position. The post hoc analysis was adjusted with the Bonferroni test.

Subsequently, Eta-squared (*η*^*2*^) was used to quantify the effect size (ES) for interpreting the differences found between groups [23]. The values (*η*^*2*^) for the ANOVA tests performed were 0.10 for small effects, 0.25 for moderate effects and 0.50 for large effects [24]. The values of effect size for two-way ANOVA (*η*^*2*^_*p*_) were 0.02 for small effects, 0.15 for moderate effects, and 0.35 for large effects. Regarding the effect size for the *t*-tests, it was interpreted with Cohen’s *d* values. For the interpretation of this effect size, the following criteria were used: small effect (*d* < 0.20), moderate effect (0.20 ≤ *d* < 0.80) and large effect (*d* ≥ 0.80) [25]. The significance level was set at *p* < .05.

## Results

Table 2 showed the physical performance metrics during competitive matches according to the playing role (starters and non-starters). Significant differences were observed in the distance covered (m), number of accelerations and decelerations, explosive distance covered (m), metabolic power (W/kg) and stay/walk. In all these metrics, the starter players showed higher demands than the non-starter players, obtaining a large effect size. However, no differences were found with the relative data.

**Table 2:** *T*-test of the physical performance metrics between starters and non-starters players

		Starter players (n = 16)			Non-starter players (n = 16)			*t*	*p*	*d*
Absolute data	Distance (m)	6644.9	±	1616.2	3228.3	±	1121.4	4.913	0.000	2.46
	Accelerations (count)	1987.0	±	459.1	968.1	±	282.3	5.347	0.000	2.67
	Decelerations (count)	1993.5	±	456.3	973.1	±	277.0	5.407	0.000	2.70
	Explosive distance (m)	601.5	±	155.1	280.1	±	103.2	4.879	0.000	2.44
	Player Load (a.u.)	90.5	±	25.8	46.2	±	17.0	4.058	0.002	2.03
	Power Metabolic (W/kg)	17598.6	±	4280.2	8377.2	±	2939.9	5.023	0.000	2.51
	Stay/Walk 0–6 (m)	535.9	±	151.2	212.0	±	99.4	5.063	0.000	2.53
Relative data	Distance/time (m/min)	97.2	±	10.9	95.6	±	12.2	0.274	0.788	0.14
	Accelerations (count/min)	29.9	±	1.7	29.0	±	2.8	0.793	0.444	0.39
	Decelerations (count/min)	29.3	±	2.0	29.2	±	2.9	0.134	0.895	0.04
	Explosive distance (m/min)	8.8	±	1.1	8.3	±	1.5	0.696	0.499	0.38
	LSRD (m/min)	36.9	±	17.9	39.7	±	19.5	− 0.291	0.775	− 0.15
	MDSRD (m/min)	20.8	±	7.8	21.6	±	6.4	− 0.232	0.820	− 0.11
	HSRD (m/min)	4.3	±	1.4	4.0	±	1.2	0.416	0.684	0.23
	VHSRD (m/min)	2.2	±	1.1	1.8	±	1.0	0.695	0.498	0.38
	SPD (m/min)	1.4	±	0.8	1.0	±	0.7	1.180	0.258	0.53
	Player Load (a.u./min)	1.3	±	0.2	1.4	±	0.3	− 0.387	0.705	− 0.39
	Power Metabolic (W/kg/min)	226.7	±	91.9	213.8	±	87.9	0.287	0.779	0.14

LSRD: low speed running distance covered between 6 to 12 km·h^− 1^; MSRD: medium speed running distance covered between 12 to 18 km·h^− 1^; HSRD: high-speed running distance covered between 18 to 21 km·h^− 1^; VHSRD: very high-speed running distance covered between 21 to 24 km·h^− 1^; SPD: sprint running distance covered between 24 to 50 km·h^− 1^

In addition, a one-way ANOVA test, between the physical performance metrics and the playing position as factor, was carried out (Table 3). Midfielders obtained the highest distances covered per minute, MSRD, and player load (a.u./min), and center-backs were the player that covered the lowest distances in the same metrics. However, the HSRD obtained similar records in the four playing positions, except for the center-backs with the lowest distance covered in this speed range. The full-back position had the highest distances and the midfielders the lowest SPD.

**Table 3:** One-way ANOVA test of physical performance metrics of soccer players according to the playing position

		Full-back (n = 4)	Center-back (n = 8)	Winger/Forward (n = 10)	Midfielder (n = 10)	*F*_*(3,30)*_	*p*	*η* ^*2*^
Absolute data	Distance (m)	6596.2 ± 1880.9	4781.1 ± 2477.0	3737.5 ± 2103.2	5596.3 ± 2380.9	1.018	0.419	0.203
	Explosive distance (m)	646.2 ± 103.5	412.7 ± 237.4	346.5 ± 228.9	475.5 ± 179.3	1.066	0.400	0.210
	Stay/Walk (m)	589.6 ± 113.7	352.5 ± 220.1	286.5 ± 230.9	392.3 ± 188.7	1.049	0.406	0.208
	Player Load (a.u.)	83.3 ± 0.2	63.0 ± 35.1	51.9 ± 28.2	83.0 ± 34.1	1.034	0.412	0.205
	Power Metabolic (W/kg)	17708.3 ± 808.6	12567.8 ± 6652.9	9857.7 ± 5818.7	14566.2 ± 6190.6	1.011	0.422	0.202
Relative data	Distance/time (m/min)	95.1 ± 8.4	87.7 ± 9.9 ^**Mi**^	91.8 ± 6.0 ^**Mi**^	108.6 ± 7.4 ^**Ce,Wi**^	6.301	0.008	0.612
	Accelerations (count/min)	29.5 ± 1.7	31.0 ± 2.0	29.4 ± 2.8	28.2 ± 2.1	1.030	0.414	0.205
	Decelerations (count/min)	29.5 ± 1.5	31.2 ± 1.8 ^**Mi**^	29.7 ± 2.9	27.1 ± 0.7 ^**Ce**^	3.337	0.056	0.455
	Explosive distance (m/min)	9.2 ± 0.4	7.4 ± 1.4	8.2 ± 0.9	9.4 ± 1.2	2.841	0.083	0.415
	LSRD (m/min)	26.3 ± 4.0	40.5 ± 25.1	31.2 ± 6.3	48.4 ± 21.2	1.112	0.382	0.218
	MDSRD (m/min)	20.0 ± 4.6	14.8 ± 5.6 ^**Mi**^	20.3 ± 3.4	27.7 ± 6.5 ^**Ce**^	4.686	0.022	0.539
	HSRD (m/min)	4.7 ± 0.8	2.7 ± 0.9 ^**Mi**^	4.6 ± 1.1	4.7 ± 1.0 ^**Ce**^	3.666	0.044	0.478
	VHSRD (m/min)	3.3 ± 0.7	1.3 ± 0.8	2.3 ± 0.9	1.8 ± 1.1	2.318	0.127	0.367
	SPD (m/min)	2.4 ± 0.1 ^**Mi**^	1.0 ± 0.5	1.6 ± 0.4	0.6 ± 0.6 ^**Fu**^	6.885	0.006	0.633
	Player Load (a.u./min)	1.2 ± 0.1 ^**Mi**^	1.1 ± 0.2 ^**Mi**^	1.3 ± 0.1 ^**Mi**^	1.6 ± 0.1 ^**Fu,Ce,Wi**^	8.195	0.003	0.672
	Power Metabolic (W/kg/min)	254.9 ± 18.3	168.6 ± 109.0	239.9 ± 20.1	228.1 ± 123.4	0.622	0.614	0.135

LSRD: low speed running distance covered between 6 to 12 km·h^− 1^; MSRD: medium speed running distance covered between 12 to 18 km·h^− 1^; HSRD: high-speed running distance covered between 18 to 21 km·h^− 1^; VHSRD: very high-speed running distance covered between 21 to 24 km·h^− 1^; SPD: sprint running distance covered between 24 to 50 km·h^− 1^. ANOVA test (*p* < .05); Bonferroni Post hoc test: Fu: Differences with full-backs; Ce: Differences with center-backs; Wi: Differences with winger/forward; Mi: Differences with midfielder

Thus, the influence between the physical metrics highlighted and the role and playing position were analyzed through two-way ANOVA test. Table 4 shows the values of different physical performance metrics of the players obtained during the competition. It is obtained that the SPD variable is influenced by role and playing position (*R*^2^adj = 0.88; *p* < .001; *η*_*p*_^*2*^ = 0.772).

**Table 4:** Two-way ANOVA test of the physical performance demands of soccer players according to playing position and playing role

Variable	*F*	R^2^ adj	*p*	*η*_*p*_^*2*^
SPD (m/min)	19.487	0.881	0.001	0.772
VHSRD (m/min)	1.860	0.256	0.268	0.254
HSRD (m/min)	2.314	0.345	0.956	0.010
MDSRD (m/min)	2.162	0.317	0.724	0.069
LSRD (m/min)	0.749	− 0.111	0.496	0.144
Explosive distance (m/min)	2.573	0.386	0.231	0.278
Explosive distance (m)	5.379	0.637	0.513	0.138
Decelerations (count/min)	1.761	0.233	0.535	0.130
Accelerations (count/min)	0.928	− 0.029	0.341	0.213
Distance/time (m/min)	3.471	0.497	0.574	0.116
Decelerations (count)	4.212	0.562	0.444	0.165
Accelerations (count)	4.197	0.561	0.428	0.172
Player Load (a.u.)	5.302	0.632	0.850	0.036
Player Load (a.u./min)	4.002	0.546	0.567	0.119
Power Metabolic (W/kg)	5.288	0.632	0.792	0.050
Power Metabolic (W/kg/min)	0.520	− 0.238	0.599	0.108
Stay/Walk 0–6 (m)	5.465	0.641	0.470	0.154

LSRD: low speed running distance covered between 6 to 12 km·h^− 1^; MSRD: medium speed running distance covered between 12 to 18 km·h^− 1^; HSRD: high-speed running distance covered between 18 to 21 km·h^− 1^; VHSRD: very high-speed running distance covered between 21 to 24 km·h^− 1^; SPD: sprint running distance covered between 24 to 50 km·h^− 1^

## Discussion

The aim of this study was to analyze and compare different physical performance metrics during competitive matches, depending on the players role (starters or non-starters) and on the playing position. The main finding of this study showed that substitute players did not develop a different physical performance than starter players, analyzing the metrics in relative values. In this aspect, in the scientific literature, there are few contributions of studies that try to analyze the performance profile of non-starter players in competition. Thus, in modern soccer, where there are increasingly greater physical demands, it becomes more important for non-starter players to be able to offer an efficient sports performance, at any period of the season. One study of a professional team’s season observed that a conditioning factor for the team to win the championship was a greater contribution of goals by non-starter players, related to sports success and increasing the importance of these players throughout the season [26].

The published studies included all substitutions made during the competition, regardless of the reason and the time played [9, 27]. In the present study, the substitutions were analyzed when the players managed to play a minimum of 15 min. In this sense, taking into account the inclusion criteria, 21 substitutions were deleted. This reason was mainly due to the loss of time due to a favorable score, in most cases, and in addition, it was thought that substitutes who start from the 75th minute of play would have a higher intensity of play compared to those who have played all the rest and have accumulated a very high level of fatigue. So, several studies about the performance of non-starter players, demonstrated that these players were able to increase the performance of the team and were determinant for sports success [9].

Non-starter players introduced during matches have achieved higher performance in different physical metrics, compared to players who were substituted and those who completed the full match [9, 28–30]. In this study, significant differences in absolute physical metrics were found, e.g., total distance covered (m), with the non-starter players presenting lower demands than the starter players, justified by a higher playing time in the match by the starter players [28, 31]. Therefore, we focus on the results of relative metrics as a function of playing time, which will provide us with objective data on player performance. In the case of the relative distance covered, the data showed very similar distances covered between both groups, starters (97.2 ± 10.9) and non-starters (95.6 ± 12.2). These data were not consistent with those found in other similar study, since the non-starter players in professional teams obtained greater distances covered in meters per minute [29].

On the one hand, accelerations and decelerations were actions that influenced the game, which increased the metabolic demands of the players, being a measure of load for the player [32]. In this study, these metrics of accelerations and decelerations per minute showed hardly any differences between the two groups. However, HSRD was a variable to be considered in sports performance. It is a key indicator of sports success in professional soccer, as many decisive moments are marked by explosive and/or high intensity or speed actions [5]. In the present study, no significant differences were obtained, resulting in very similar data for running at different speeds. Starters and non-starters obtained similar values in these speed ranges (HSRD; 18 to 21 km·h^− 1^); and very high speed (VHSRD; 21 to 24 km·h^− 1^) and sprint (SPD; 24 to 50 km·h^− 1^). Thus, non-starters obtained the similar performance than starters [9, 30]. In addition, these results showed the physical performance of players has implied in the substitutions, so these findings allow compare only the players implied, deleting the other players that play all the match. However, non-starter players played the match lower time and may not dose their efforts, as starter players must do strategically, in order to dose their efforts for all match situations, i.e., total distance covered per minute, distance covered higher than 21 km·h^− 1^, accelerations, among others [33]. In this sense, this variable of intensity seems to be of great importance, since one of the main reasons for substitutions is to increase the performance of the team and that this has a physical impact, therefore, this variable would help the coach to quantify the maximum efforts that the non-starter player can offer and thus, evaluate the substitution or choose those players who will offer a higher performance [16, 28].

On the other hand, this study analyzed whether the playing position influences the main physical performance metrics, obtaining significant results [30]. Non-starter attacking players recorded greater high-intensity distances compared to their teammates when completing the entire match [9]. In terms of relative distance covered, the midfielders were the players who covered the most distance per minute and center-backs the least. Thus, midfielders are the players who cover the greatest distances among the starters, but similarly, non-starter players in this position cover greater distances than their respective non-starters in other positions [28, 34]. In the MDSRD and HSRD metrics, midfield players performed greater distances than other playing positions too. The SPD metric has shown differences between the playing positions. The full-back players have shown the highest records, followed by the wingers and forwards [9, 35]. Therefore, the relative distance covered is dependent of the playing position. Through these findings, it can establish the demands of each playing position and the performance that must be offered by the non-starter and starter players. It can also establish that the playing position could influence the SPD metric, which, as mentioned above, was an indicator of success in soccer. Regarding the player load variable (a.u./min), midfield players were again the ones with the highest load records, related to the fact that they cover greater distances and distances at medium and high speed [9].

As limitations of the present study, firstly, the sample size was small and should be increased to achieve more representative results for this category of soccer players. However, this sample represents the high-level of the expertise, due to the team being ranked in the top 4 of the group. In addition, the number of available GPS devices was limited, as it was not possible to quantify all the data of all the starter and non-starter players in the squad, in this case the coach’s decisions conditioned the measurement of the data, as the uncertainty of the matches did not allow for those players who were going to be substituted or introduced in the match. Future researchers are encouraged to propose studies in which the coach could choose a real reason for each change, such as loss of performance of a player, tactical change, lose time, among other reasons. On the other hand, there were factors that should be taken into account for future research, as the performance of these non-starter players can be affected by different contextual metrics, such as: whether they are playing at home or away, the level of the opposition, the stage of the season, the score at which the non-starter player enters, the minute at which he enters, as well as the mental situation of the player, all of which can be the subject of future research. Finally, there is scarce research on the performance of non-starter soccer players and therefore, the comparison of data and conclusions becomes more difficult.

## Conclusion

In conclusion, the main finding of this study showed that non-starter did not present an improvement or deterioration in physical performance in the main physical metrics analyzed, compared to starter players. This study has been based exclusively on substitutions made with at least 15 min of participation by non-starters. Additionally, playing position has been shown to influence some of the important metrics in a team’s performance.

As practical applications, coaches who substitute players at various times during the match, where the reason is focused on improved intensity or increased physical performance, must still consider the type of contribution a particular substitute could make based on situational, technical, and positional factors and also the specific moment. It seems that, from the analysis of other similar studies where the totality of substitutions had been investigated, regardless of the timing, the performance of the non-starters was superior in relative values. This information could be beneficial to coaches in optimizing their players’ performance during the match.

## Data Availability

The datasets generated during and analyzed during the current study are available from the corresponding author on reasonable request.

## Abbreviations

LSRD: Low speed running distance, from 6 to 12 km·h^− 1^
MSRD: Medium speed running distance, from 12 to 18 km·h^− 1^
HSRD: High-speed running distance, from 18 to 21 km·h^− 1^
VHSRD: Very high-speed running distance, from 21 to 24 km·h^− 1^
SPD: Sprint running distance, from 24 to 50 km·h^− 1^
